# SARS-CoV-2 Seroprevalence before Delta Variant Surge, Chattogram, Bangladesh, March–June 2021

**DOI:** 10.3201/eid2802.211689

**Published:** 2022-02

**Authors:** Taufiqur Rahman Bhuiyan, Juan Dent Hulse, Sonia T. Hegde, Marjahan Akhtar, Taufiqul Islam, Zahid Hasan Khan, Ishtiakul Islam Khan, Shakeel Ahmed, Mamunur Rashid, Rumana Rashid, Emily S. Gurley, Tahmina Shirin, Ashraful Islam Khan, Andrew S. Azman, Firdausi Qadri

**Affiliations:** icddr,b, Dhaka, Bangladesh (T.R. Bhuiyan, M. Akhtar, T. Islam, Z.H. Khan, I.I. Khan, A.I. Khan, F. Qadri);; Johns Hopkins Bloomberg School of Public Health, Baltimore, Maryland, USA (J.Dent. Hulse, S.T. Hegde, E.S. Gurley, A.S. Azman);; Bangladesh Institute of Tropical and Infectious Diseases, Chattogram, Bangladesh (S. Ahmed, M. Rashid, R. Rashid);; Institute of Epidemiology, Disease Control and Research, Dhaka (T. Shirin);; Institute of Global Health, University of Geneva, Geneva, Switzerland (A.S. Azman)

**Keywords:** COVID-19, Delta variant, coronavirus disease, severe acute respiratory syndrome coronavirus 2, SARS-CoV-2, seroepidemiologic studies, respiratory infections, zoonoses, Bangladesh

## Abstract

A March–June 2021 representative serosurvey among Sitakunda subdistrict (Chattogram, Bangladesh) residents found an adjusted prevalence of severe acute respiratory syndrome coronavirus 2 antibodies of 64.1% (95% credible interval 60.0%–68.1%). Before the Delta variant surge, most residents had been infected, although cumulative confirmed coronavirus disease incidence was low.

Through November 9, 2021, Bangladesh had reported >1.57 million COVID-19 cases and 27,904 deaths (*1*), with incidence and mortality rates substantially lower than in many other countries. Without performing population-based seroprevalence estimates, it is difficult to know whether differences in rates of illness and death result from undercounts because of limited surveillance and healthcare seeking or reflect actual differences in incidence resulting from interventions or different biological responses to infection. In early March 2021, cases across Bangladesh began to rise at the same time as the Delta variant was detected in neighboring India. Publicly available sequencing data (*2*) indicate that the SARS-CoV-2 Delta variant was first detected in the Chattogram region of Bangladesh in mid-May 2021, and 99% (98/99) of the viral genomes submitted during July 1–October 1, 2021 have been of the Delta variant, similar to national trends.

## The Study

We conducted a representative serosurvey to understand the prevalence of total SARS-CoV-2 antibodies in residents of the Sitakunda subdistrict (Chattogram district) of Bangladesh, a region with an urban-to-rural gradient that includes Chattogram, Bangladesh’s second largest city. We conducted the survey over 2 periods, March 27–April 13 and May 23–June 13, because of a national COVID-19 lockdown (April 14–May 30). We used 2-stage sampling based on digitized satellite imagery by first dividing the Sitakunda subdistrict into 1 km^2^ grid-cells (or clusters) and randomly selecting grid-cells proportional to the estimated number of households in each, with replacement. We then randomly selected structures weighted by whether they were multistory or single-story. We attempted to enroll all persons ≥1 year of age in each household.

We tested participant serum for total antibodies (IgA, IgM, and IgG) against the receptor-binding domain of SARS-CoV-2 using the SARS-CoV-2 Ab ELISA (Wantai BioPharm, https://www.ystwt.cn), following manufacturer instructions. We corrected seroprevalence estimates for imperfect test performance, household clustering, and individual-level covariates (e.g., age) using a Bayesian modeling approach documented elsewhere and stratified results to match the target population (*3*). Our study was approved by the icddr,b research and ethics review committee and the Johns Hopkins Bloomberg School of Public Health institutional review board.

Given limited data on the immunoassay’s performance in south Asia and performance months after infection, we conducted a validation study to estimate its sensitivity and specificity by testing samples from 214 healthy participants from a 2014 cholera vaccine study and 81 from 52 symptomatic PCR-confirmed SARS-CoV-2–infected patients; none of the positive controls had been hospitalized or vaccinated for COVID-19. We collected samples 3–275 days after symptom onset. We estimated specificity at 99.1% (95% CI 96.7%–99.9%, n = 212/214) and sensitivity at 87.7% (95% CI 78.5%–93.9%, n = 71/81) for detecting previous infection with little evidence of sensitivity decreasing over time after infection (Appendix Table 4).

We enrolled 580 households and 2,307 participants who provided a blood sample. Most participants (54%, n = 1,235/2,307) were female and the median age was 28 (interquartile ratio 16–45) years; most reported working at home (37%), going to school (29%), or conducting business outside of their home (20%) as their main occupation in the month before enrollment. Among all participants, 22 (0.95%) reported ever having a COVID-19 test; 3/22 had positive results (all 3 were also seropositive in the study). Of 2,307 participants, 125 (5.4%) reported being vaccinated (15–144 days before interview) with >1 dose of SARS-CoV-2 vaccines, including 117 with a CoviShield ChAdOx1 (Serum Institute of India, https://www.seruminstitute.com) vaccination card and 1 with a Pfizer/BioNTech BNT162n2 (https://www.pfizer.com) vaccination card. As of June 19, 2021, 6 days after the end of the survey, 6.0% of the entire Chattogram district population was reported to have received >1 dose of any vaccine; 4.6% had received 2 doses (*4*). 

There were 1,443 (63%) seropositive participants. Nearly all (98%) who reported having been partially (47/49) or completely vaccinated (75/76) were seropositive. In 85% of enrolled households, >1 participant was seropositive and an average of 62% of participants in each household were seropositive. We estimated that 31% of the total variability in seropositivity in the community was attributable to variation in seropositivity between households (intraclass correlation coefficient 0.31, 95% CI 0.27–0.36). We found evidence of a gradient in seropositivity associated with population density. Participants living in higher population density areas were significantly more likely to be seropositive: 69% of participants living in the most population-dense areas were seropositive compared with 52% of participants living in the least population-dense areas (p<0.0001; Appendix Table 1). We found similar results using alternative metrics related to urbanicity (Appendix Table 1). Among seropositive participants, 57% (815/1,442) reported having had >1 COVID-consistent symptom since April 2020 and 58% (474/812) of these participants reported seeking healthcare.

Adjusting for age, sex, household clustering, and test performance, we estimated the seroprevalence of SARS-CoV-2 in Sitakunda to be 64.1% (95% credible interval [CrI] 60.0%–68.1%) among all participants and 63.4% (95% CrI 59.2%–67.6%) when considering only unvaccinated participants (Table; Appendix Table 3). We estimated a 7% (95% CrI 1%–13%) higher risk of being seropositive in men compared with women. Risk generally increased with age, with those <10 years of age having the lowest risk, including a >34% lower risk of being seropositive compared with those 25–34 years of age (Table; Appendix Table 3). We found similar adjusted seroprevalences in the population recruited before the lockdown (63.1%, 95% CrI 56.2%–69.8%; n = 665) and after the lockdown (65.3%, 95% CrI 60.6%–69.9%; n = 1,643). In between the 2 survey rounds, during the lockdown, the number of clinical cases district-wide decreased and, likely as a result of the Delta variant, began to increase during the end of the second round of data collection (*4*).

**Table:** Overview of SARS-CoV-2 seropositivity, seroprevalence and relative risk seropositivity in Sitakunda subdistrict, Chattogram district, Bangladesh*

Variable	Observations, no.	Positive, no. (%)	Negative, no. (%)	Adjusted seroprevalence, % (95% CrI)	Adjusted relative risk, % (95% CrI)
Age, y					
1–4	90	37 (41.1)	53 (58.9)	47.1 (37.0–57.3)	0.66 (0.51–0.81)
5–9	174	71 (40.8)	103 (59.2)	45.0 (37.1–52.9)	0.63 (0.51–0.74)
10–14	258	140 (54.3)	118 (45.7)	58.8 (52.0–65.3)	0.83 (0.73–0.94)
15–24	482	305 (63.3)	177 (36.7)	67.2 (61.7–72.6)	0.96 (0.88–1.05)
25–34	381	258 (67.7)	123 (32.3)	69.7 (64.5–75.0)	Referent
35–44	325	225 (69.2)	100 (30.8)	74.0 (68.3–79.5)	1.07 (0.97–1.17)
45–54	250	180 (72.0)	70 (28.0)	73.8 (67.2–80.3)	1.06 (0.96–1.17)
55–64	208	132 (63.5)	76 (36.5)	69.0 (62.1–75.8)	0.99 (0.88–1.10)
>65	139	95 (68.3)	44 (31.7)	73.6 (65.8–81.1)	1.06 (0.94–1.19)
Sex					
M	1,072	690 (64.4)	382 (35.6)	66.7 (62.2–71.3)	1.07 (1.02–1.13)
F	1,235	753 (61.0)	482 (39.0)	61.3 (56.9–65.6)	Referent
Overall	2,307	1,443 (62.5)	864 (37.5)	64.1 (60.0–68.1)	NA
*Adjusted estimates account for sex, age, household clustering, and test performance among all vaccinated and unvaccinated participants. CrI, credible interval; NA, not applicable.					

In the catchment area of this serosurvey, only 1 healthcare facility (Bangladesh Institute of Tropical and Infectious Diseases) provided SARS-CoV-2 PCR testing. Among the 2,400 participants who had a reverse transcription PCR test during April 2020–May 31, 2021, a total of 705 (29%) tested positive. By crudely extrapolating our serologic estimates by multiplying the estimated population size by the adjusted seroprevalence among those who were unvaccinated, we estimated that >200,000 infections occurred during the same period in Sitakunda. Assuming all positive cases were from Sitakunda and not neighboring areas, this corresponds to a minimum of 300 infections per medically confirmed case, a much higher proportion than has been documented in most settings across the world (*5*,*6*).

## Conclusions

These results illustrate that prior to the June 2021 surge in COVID-19 cases in Bangladesh fueled by the Delta variant, most of the population in Sitakunda had already been infected despite a relatively low incidence of reported virologically confirmed SARS-CoV-2 infections. Key limitations to these results include the relatively small geographic area covered by the survey and that we only assessed circulating antibodies to a single SARS-CoV-2 epitope, which does not fully capture the immune profile of participants. 

In Bangladesh, where cases captured by surveillance are limited by healthcare seeking, even in population-dense settings, representative seroprevalence surveys can help with continuing to track the evolution of this pandemic. In addition to providing important validation data on a widely used immunoassay, our results help lay the foundation for understanding the role of variant strains on key epidemiologic parameters, including our understanding of reinfection, and help set expectations for SARS-CoV-2 control in the months to come, in the study area and beyond. 

This article was preprinted at https://doi.org/10.1101/2021.07.16.21260611.

Appendix Additional information on serosurvey of SARS-CoV-2 in Chattogram, Bangladesh
