# Supplementary material for: SARS-CoV-2 Seroprevalence before Delta Variant Surge, Chattogram, Bangladesh, March–June 2021
Source: Emerg Infect Dis. 2022 Feb;28(2):429–31. doi: 10.3201/eid2802.211689 (PMC8798688; doi:10.3201/eid2802.211689)

Article DOI: https://doi.org/10.3201/eid2802.211689

SARS-CoV-2 Seroprevalence before Delta Variant Surge, Chattogram, Bangladesh, March–June 2021

Appendix

Appendix Table 1. Descriptive statistics for 2,307 serosurvey participants in Sitakunda Upazila by seropositivity*

Characteristics†	Negative, no. (%)	Positive, no. (%)	Total	P value
N	864	1,443	2,307	NA
Sociodemographic				
Age, median (range)	23 (1–92)	31 (1–97)	28 (1–97)	NA
Age, y				<0.0001
1–4	53 (59)	37 (41)	90	
5–9	103 (59)	71 (41)	174	
10–14	118 (46)	140 (54)	258	
15–24	177 (37)	305 (63)	482	
25–34	123 (32)	258 (68)	381	
35–44	100 (31)	225 (69)	325	
45–54	70 (28)	180 (72)	250	
55–64	76 (37)	132 (63)	208	
≥65	44 (32)	95 (68)	139	
Sex				1.0
M	482 (39)	753 (61)	1,235	
F	382 (36)	690 (64)	1,072	
Main activity in previous mo				0.2
Business outside home	137 (30)	319 (70)	456	
Child	54 (56)	42 (44)	96	
Farmer	34 (42)	46 (57)	80	
Homemaker	293 (35)	555 (65)	848	
Not worked (adult)	30 (42)	41 (58)	71	
Other	20 (24)	65 (76)	85	
Student	296 (44)	374 (56)	670	
Highest educational attainment				<0.0001
No schooling	167 (44)	209 (56)	376	
Primary	304 (42)	413 (58)	717	
Lower secondary	238 (35)	446 (65)	684	
Upper secondary	125 (30)	285 (70)	410	
Bachelors	24 (26)	67 (74)	91	
Postgraduate	4 (15)	23 (85)	27	
Household monthly income‡, USD				0.6
<12	0 (0)	0 (0)	0	
12–35	22 (25)	66 (75)	88	
35–59	21 (46)	25 (54)	46	
59–83	18 (36)	32 (64)	50	
83–118	79 (42)	107 (58)	186	
118–236	348 (38)	559 (62)	907	
>236	376 (37)	654 (63)	1,030	
Household size‡, median (range)	5 (2–18)	5 (1–18)	5 (1–18)	NA
Measures of urbanicity				
Friction§,¶,#				<0.0001
0.001–0.0012	166 (31)	371 (69)	537	
0.0012–0.0014	210 (36)	374 (64)	584	
0.0014–0.0018	189 (32)	410 (68)	599	
0.0018–0.0023	299 (51)	288 (49)	587	
Population density, per 1 km^2††,‡‡				<0.0001
517–2,354	250 (48)	273 (52)	523	
2,354–3,708	237 (39)	371 (61)	608	

Characteristics†	Negative, no. (%)	Positive, no. (%)	Total	P value
3,708–5,382	178 (34)	349 (66)	527	
5,382–11,360	199 (31)	450 (69)	649	
Household distance from Chittagong port**, m				<0.001
7,799–15,367	178 (32)	375 (68)	553	
15,367–26,595	212 (36)	375 (64)	587	
26,595–37,003	222 (39)	348 (61)	570	
37,003–46,579	252 (42)	345 (58)	597	
COVID-19–related factors				
Symptoms in previous mo§§	54 (34)	107 (66)	161	NA
Symptoms after 14 Apr 2020				0.2
0	374 (37)	627 (63)	1,001	
1	120 (36)	211 (64)	331	
2	166 (36)	294 (64)	460	
3	138 (39)	216 (61)	354	
4	46 (41)	65 (59)	111	
5	8 (26)	23 (74)	31	
6	4 (80)	1 (20)	5	
7	2 (29)	5 (71)	7	
Doctor or hospital care for symptoms after 14 Apr 2020				0.2
No	228 (40)	344 (60)	571	
Yes	264 (36)	474 (64)	738	
COVID-19 testing and vaccination				
Ever tested for COVID				0.3
No	857 (38)	1,427 (62)	2,284	
Once	6 (29)	15 (71)	21	
Multiple times	1 (100)	0 (0)	1	
Test result				0.2
Negative	6 (33)	12 (67)	18	
Positive	0 (0)	3 (100)	3	
Inconclusive	0 (0)	0 (0)	0	
Received COVID-19 vaccine¶¶				<0.0001
No	861 (39)	1,320 (61)	2,181	
1 dose	2 (4.1)	47 (96)	49	
2 doses	1 (1.3)	75 (99)	76	
Unknown	0 (0)	1 (100)	1	
Vaccine type				<0.0001
CoviShield/ChAdOx1	2 (1.7)	115 (98)	117	
Pfizer	0 (0)	1 (100)	1	
COVID-19–related behaviors after 14 Apr 2020				
Mask use				1.0
No	274 (40)	413 (60)	687	
Yes	589 (36)	1,030 (64)	1,619	
Mask frequency in previous week				0.5
Never	9 (50)	9 (50)	18	
1–2 times	92 (35)	173 (65)	265	
3–5 times	120 (40)	178 (60)	298	
Almost every day	366 (35)	667 (65)	1,033	
Public transportation use change##				0.2
No change	62 (50)	63 (50)	125	
Less use	345 (34)	672 (66)	1,017	
1–2 more times per day	0 (0)	0 (0)	0	
3–5 more times per day	1 (50)	1 (50)	2	
Continued change in public transportation use	335 (34)	663 (66)	998	NA

*NA, not applicable
†Chi-squared tests for trend were performed on categorical variables with numerically increasing categories and Pearson chi-squared tests of homogeneity were performed on all other categorical variables.
‡Household level proportions.
§Data from the Malaria Atlas Project (https://malariaatlas.org).
¶Sampling cluster level measures (1km^2).
#Minutes to travel 1 m.
**Lower no. = more urban.
††Data sourced from WorldPop (https://www.worldpop.org).
‡‡Lower no. = more rural.
§§COVID-like symptoms include: fever, cough, shortness of breath, loss of taste/smell, nausea, diarrhea, and vomiting.
¶¶Only 245 participants confirmed their vaccination status, whether having received it or not, by vaccination card. 94% (117/125) of participants that reported ≥1 dose of vaccination confirmed their vaccination status by a vaccination card.
##Change compared to use before April 14th, 2020. This field is restricted to only those who said they used public transport before April 14, 2020.

Appendix Table 2. Descriptive statistics for unvaccinated, serosurvey participants (n = 2,181) by seropositivity in Sitakunda Upazila. This table includes sociodemographic factors, measures of urbanicity, COVID-like symptoms, COVID testing and vaccination, and COVID-19–related behaviors.

Characteristics†	Negative, no. (%)	Positive, no. (%)	Total, no.	P value
N	268	371	2,181	NA
Sociodemographic				
Age, median (range)	23 (1–92)	29 (1–97)	26 (1–97)	NA
Age, y				<0.0001
1–4	53 (59)	37 (41)	90	
5–9	103 (59)	71 (41)	174	
10–14	118 (46)	140 (54)	258	
15–24	176 (37)	303 (63)	479	
25–34	123 (33)	249 (67)	372	
35–44	100 (34)	198 (66)	298	
45–54	69 (32)	147 (68)	216	
55–64	76 (44)	97 (56)	173	
≥65	43 (36)	78 (64)	121	
Sex				1.0
M	480 (41)	700 (59)	1,180	
F	381 (38)	620 (62)	1,001	
Main activity in previous mo				0.2
Business outside home	136 (32)	284 (68)	420	
Child	54 (56)	42 (44)	96	
Farmer	34 (45)	42 (55)	76	
Homemaker	291 (37)	494 (63)	785	
Not worked (adult)	30 (49)	31 (51)	61	
Other	20 (27)	53 (73)	73	
Student	296 (44)	373 (56)	669	
Highest educational attainment				<0.0001
No schooling	166 (45)	199 (55)	365	
Primary	302 (44)	392 (56)	694	
Lower secondary	238 (37)	414 (63)	652	
Upper secondary	125 (34)	248 (66)	373	
Bachelors	24 (32)	51 (68)	75	
Postgraduate	4 (20)	16 (80)	20	
Household monthly income‡, USD				0.5
<12	0 (0)	0 (0)	0	
12–35	22 (28)	58 (72)	80	
35–59	21 (49)	22 (51)	43	
59–83	18 (36)	32 (64)	50	
83–118	78 (43)	102 (57)	180	
118–236	347 (40)	517 (60)	864	
>236	375 (39)	589 (61)	964	
Household size‡, median (range)	5 (2,18)	6 (1,18)	5 (1,18)	NA
Measures of urbanicity				
Friction§,¶,#				<0.0001
0.001–0.0012	165 (33)	331 (67)	496	
0.0012–0.0014	209 (38)	348 (62)	557	
0.0014–0.0018	189 (34)	375 (66)	564	
0.0018–0.0023	298 (53)	266 (47)	564	
Population density, per 1 km^2††,‡‡				<0.0001
517–2,354	249 (50)	250 (50)	499	
2,354–3,708	236 (40)	347 (60)	583	
3,708–5,382	178 (36)	313 (64)	491	
5,382–11,360	198 (33)	410 (67)	608	
Household distance from Chittagong port**, m				<0.001
7,799–15,367	177 (35)	336 (65)	513	
15,367–26,595	212 (37)	355 (63)	567	
26,595–37,003	221 (40)	325 (60)	546	
37,003–46,579	251 (45)	304 (55)	555	
COVID-19–related factors				
Symptoms in previous mo§§	54 (36)	97 (64)	151	NA
Symptoms after 14 Apr 2020				0.4
0	372 (39)	570 (61)	942	
1	120 (38)	197 (62)	317	
2	166 (38)	266 (62)	432	
3	138 (41)	200 (59)	338	
4	46 (43)	60 (57)	106	

Characteristics†	Negative, no. (%)	Positive, no. (%)	Total, no.	P value
5	8 (28)	21 (72)	29	
6	3 (75)	1 (25)	4	
7	2 (33)	4 (67)	6	
Doctor or hospital care for symptoms after 14 Apr 2020				1.0
No	228 (41)	321 (59)	549	
Yes	263 (38)	432 (62)	695	
COVID-19 testing and vaccination				
Ever tested for COVID				0.5
No	854 (40)	1,308 (60)	2,162	
Once	6 (35)	11 (65)	17	
Multiple times	1 (100)	0 (0)	1	
Test result				0.2
Negative	6 (43)	8 (57)	14	
Positive	0 (0)	3 (100)	3	
Inconclusive	0 (0)	0 (0)	0	
COVID-19–related behaviors after 14 Apr 2020				
Mask use				1.0
No	272 (41)	385 (59)	657	
Yes	588 (39)	935 (61)	1,523	
Mask frequency in the previous week				0.9
Never	9 (53)	8 (47)	17	
1–2 times	92 (36)	163 (64)	255	
3–5 times	120 (41)	170 (59)	290	
Almost every day	365 (38)	591 (62)	956	
Public transportation use change¶¶				0.2
No change	61 (51)	58 (49)	119	
Less use	344 (37)	598 (63)	942	
1–2 more times per day	0 (0)	0 (0)	0	
3–5 more times per day	1 (50)	1 (50)	2	
Continued change in public transportation use	334 (36)	590 (64)	924	NA

*NA, not applicable
†Chi-squared tests for trend were performed on categorical variables with numerically increasing categories and Pearson chi-squared tests of homogeneity were performed on all other categorical variables.
‡Household level proportions.
§Data from the Malaria Atlas Project (6).
¶Sampling cluster level measures (1km^2).
#Minutes to travel 1 m.
**Lower no. = more urban.
††Data sourced from WorldPop (https://www.worldpop.org).
‡‡Lower no. = more rural.
§§COVID-like symptoms include: fever, cough, shortness of breath, loss of taste/smell, nausea, diarrhea, and vomiting.
¶¶Change compared to use before April 14th, 2020. This field is restricted to only those who said they used public transport before April 14, 2020.

Appendix Table 3. Estimated seroprevalence of SARS-CoV-2 in Sitakunda Upazila adjusted for sex, age, household clustering, and test performance among unvaccinated participants.

Variable	Observations	Positive	Negative	Adjusted seroprevalence (95% CI)	Adjusted relative risk (95% CI)
Age					
1–4 y	90	37 (41.1)	53 (58.9)	47.3 (37.4–57.5)	0.67 (0.53–0.82)
5–9 y	174	71 (40.8)	103 (59.2)	44.8 (37.1–52.9)	0.63 (0.52–0.74)
10–14 y	258	140 (54.3)	118 (45.7)	59.2 (52.3–66.2)	0.85 (0.74–0.95)
15–24 y	479	303 (63.3)	176 (36.7)	67.6 (62.2–73.1)	0.98 (0.89–1.07)
25–34 y	372	249 (66.9)	123 (33.1)	69.1 (63.7–74.3)	Referent
35–44 y	298	198 (66.4)	100 (33.6)	72.4 (66.4–78.3)	1.05 (0.95–1.15)
45–54 y	216	147 (68.1)	69 (31.9)	71.3 (63.8–78.4)	1.03 (0.92–1.15)
55–64 y	173	97 (56.1)	76 (43.9)	63.8 (55.9–71.4)	0.92 (0.80–1.04)
≥65 y	121	78 (64.5)	43 (35.5)	71.2 (62.6–79.8)	1.04 (0.90–1.17)
Sex					
M	1001	620 (61.9)	381 (38.1)	65.8 (61.0–70.6)	1.07 (1.01–1.13)
F	1180	700 (59.3)	480 (40.7)	60.7 (56.3–65.3)	Referent
Overall	2,181	1,320 (60.5)	861 (39.5)	63.4 (59.2–67.6)	NA

Appendix Table 4. The number of positive controls used to estimate the empirical sensitivity of the Wantai total Ab assay and SARS-CoV-2 positivity by time since symptom onset.

Time post-symptom onset, d	No. samples	% Seropositive (n)
3–13	2	100 (2)
14–30	6	83.3 (5)
31–60	5	100 (5)
61–90	7	100 (7)
91–120	7	85.7 (6)
121–150	10	80 (8)
151–180	12	83.3 (10)
181–210	11	72.7 (8)
211–240	9	100 (9)
241–275	12	100 (12)

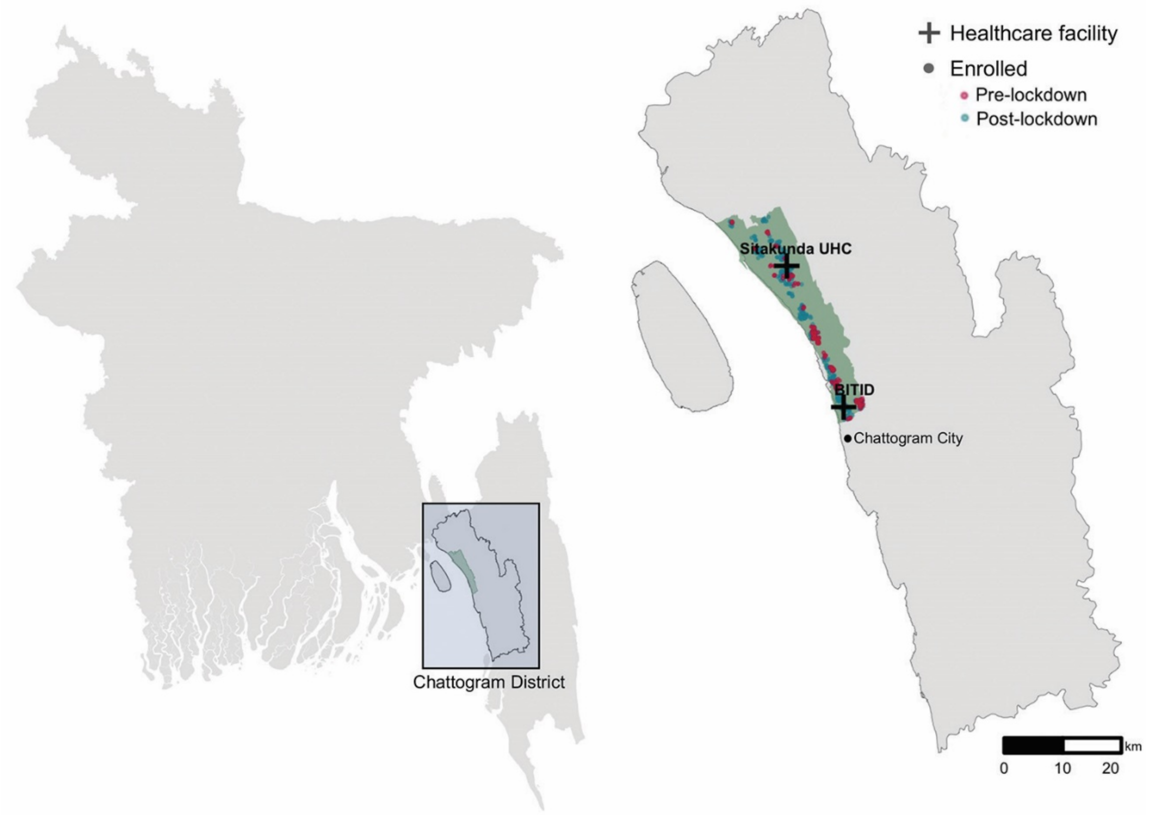

Appendix Figure 1. Map of the study population in Sitakunda Upazila (green) in the Chattogram District of Bangladesh. The 580 enrolled households sampled in the serosurvey by enrollment time (pre- versus post-lockdown) and the 2 healthcare facilities in Sitakunda (Bangladesh Institute of Tropical & Infectious Diseases and Sitakunda Upazila Health Complex) are shown on the right side of the figure.

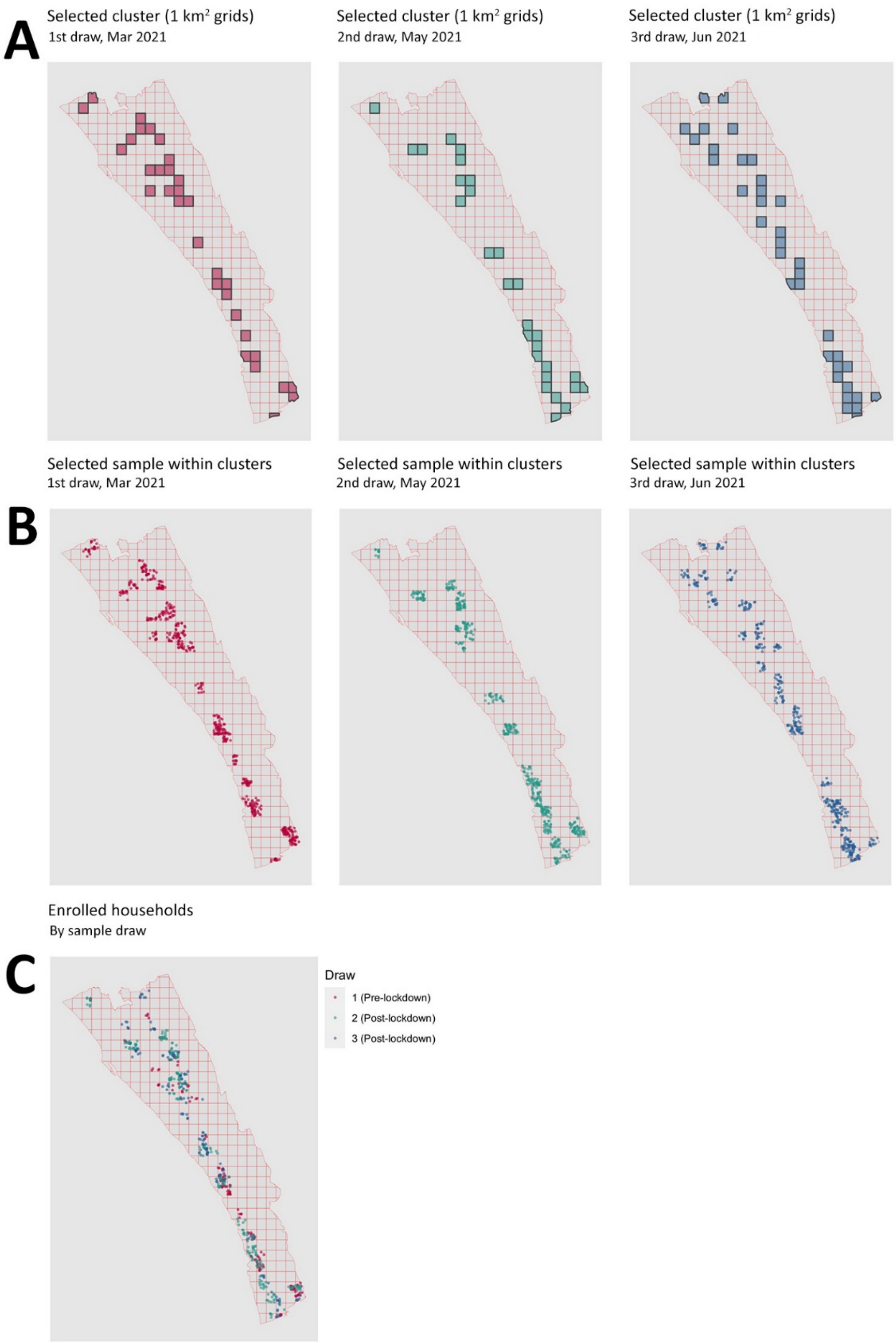

Appendix Figure 2. Map of the sampled clusters and sampled dwellings within clusters in the Sitakunda Upazila by samples drawn at three different periods: first draw (March 2021), second draw (May 2021), third draw (June 2021). Three separate sample draws were conducted because of interruption from the nationally imposed lockdown and a large percentage of nonresidential structures among housing

structures sampled from the satellite imagery. A) Clusters were sampled 41 times with 14 structures each during the first 2 draws and 12 structures during the third draw. B) We oversampled the number of structures by 40% to account for nonresidential buildings for a total of 574 sampled structures for the first 2 draws and 492 for the third draw. C) Households were enrolled across the entire subdistrict of Sitakunda during each enrollment period and by sample draw (households enrolled pre-lockdown were only drawn from the first sample).

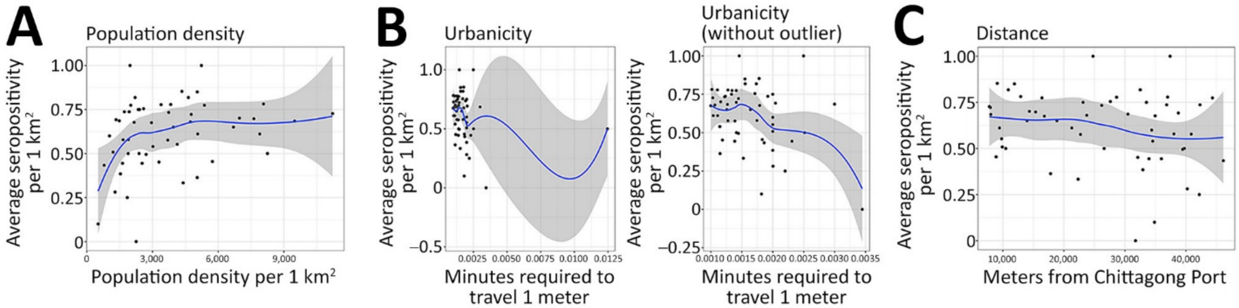

Supplement: Appendix — Additional information on serosurvey of SARS-CoV-2 in Chattogram, Bangladesh [file 21-1689-Techapp-s1.pdf]
